# Prevalence and characteristics of lumbar ribs: a meta-analysis with anatomical and clinical considerations

**DOI:** 10.1007/s00276-024-03504-9

**Published:** 2024-10-08

**Authors:** Maksymilian Osiowski, Aleksander Osiowski, Maciej Preinl, Kacper Stolarz, Tomasz Klepinowski, Barbara Jasiewicz, Dominik Taterra

**Affiliations:** 1 Faculty of Medicine, Jagiellonian University Medical College, sw. Anny 12, Krakow, 31-008 Poland; 2 Department of Orthopedics and Rehabilitation, Jagiellonian University Medical College, Balzera 15, Zakopane, 34- 500 Poland; 3 Ortho and Spine Research Group, Zakopane, Poland; 4 Department of Neurosurgery, Pomeranian Medical University Hospital, No. 1, Unii Lubelskiej 1, Szczecin, 71-252 Poland

**Keywords:** Lumbar ribs, Supernumerary ribs, Lumbar spine anomalies, Spine malformations

## Abstract

**Background:**

Lumbar ribs (LR) are a rare and relatively unknown anatomical abnormality of the lumbar spine. The literature provides better understanding regarding other spinal congenital variations like cervical ribs or lumbosacral transitional vertebrae, which are rather commonly recognised conditions. Thus, this meta-analysis aimed to provide data on prevalence and key characteristics of LR.

**Methods:**

Relevant databases were systematically searched for studies reporting the prevalence, laterality and geographic distribution of LR. No exclusion criteria based on language and date of original articles were employed. The pooled prevalence estimates (PPE) were calculated using a random-effects model. To assess the between-study heterogeneity, the *I*^*2*^ statistic and Chi-square test were utilized. Throughout the investigation, the PRISMA guidelines were adhered to scrupulously. Evaluation of the included studies’ reliability was made with the AQUA tool.

**Results:**

In total, 9 studies were included in this meta-analysis. The pooled prevalence estimate (PPE) of LR was 2.1% (95%CI: 1.0-4.6). In studies based on CT imaging, LR were found in 1.6% (95%CI: 0.6–4.3) of patients and in Xray based studies in 2.1% (95%CI: 0.4–11.1) of patients. Lumbar ribs were bilateral in majority of individuals (65.4%, 95%CI: 39.4–84.6) and could be most frequently encountered in Europe with PPE of 2.8% (95%CI: 3.0–20.0), then in East Asia with PPE of 1.5% (95%CI: 1.0-19.2) and Middle East with PPE of 1.1% (95%CI: 0.6–20.0).

**Conclusions:**

The findings of our study indicate that LR are a common anatomical variation of lumbar spine, contrary to previous beliefs. In a clinical practice, when a patient presents with a non-specific low back pain, a possible occurrence of LR should be taken into consideration. The presence of LR may be misleading for surgeons and result in wrong-level spine surgeries.

**Supplementary Information:**

The online version contains supplementary material available at 10.1007/s00276-024-03504-9.

## Introduction

Lumbar ribs (LR) are a relatively rare malformation of the lumbar spine, which can vary in form from fully developed to rudimentary, additional ribs. These ribs have also been referred to as gorilla ribs in the literature since gorillas only have four lumbar vertebrae [4]. Cumming [13] reported the first lumbar rib case in 1926. This patient’s lumbar rib originated from the third lumbar vertebra’s transverse process, descended, and fused with the fourth lumbar vertebra’s transverse process on the same side [13]. Although LR can originate from the second [34] and third vertebrae [13], they most frequently originate from the first lumbar vertebra [5]. While LR have a different course from typical ribs, they usually resemble floating ribs in terms of appearance [5]. LR typically do not create any kind of structural deformity and are predominantly asymptomatic, however, some patients may experience unpleasant symptoms related to their presence, such as pain located specifically in the renal angle region [5]. The exact pathophysiology underlying LR formation as well as data regarding their prevalence remain unknown; nevertheless, numerous researchers have suggested LR to be a result of mutations in Homebox (HOX) genes [29, 46]. Overall, rib abnormalities are believed to occur in 1% of the population [47], however, the data in the literature mainly concerns cervical ribs (CR) [23], while LR remain a rare anomaly with scarcely reported prevalence [5]. Moreover, due to their uncommonness, LR are frequently confused with osteophytes, transverse process anomalies, or even abnormalities of the abdominal vessels [28, 40], which may lead to further underestimation of their prevalence. When present and not recognized they can lead to incorrect spinal level identification during spinal surgery and as such wrong level surgery. Therefore, our analysis’s main objective was to provide a thorough, evidence-based assessment of LR prevalence, from which clinicians, especially spinal surgeons, may benefit to avoid wrong level surgery as well as when performing a differential diagnosis of patients with lower back pain of unknown origin.

## Methods

### Search strategy and information sources

Search of all major bibliographic databases (Pubmed, Embase and Web of Science) was conducted in order to identify all studies reporting relevant data on lumbar ribs up to June 2024. The following search terms were applied: lumbar rib OR supernumerary rib OR extra rib OR accessory rib OR thoracolumbar transitional vertebrae OR TLTV OR gorilla rib OR accessory ossification center. No language or date restrictions were imposed. Additionally the references of every initially selected study were analyzed to make sure no eligible articles are omitted. Authors strictly adhered to the Preferred Reporting Items for Systematic Reviews and Meta-Analyses (PRISMA) guidelines while conducting this study [36] (Supplementary material 1).

### Eligibility criteria and study selection process

Relevant studies reporting on the prevalence and characteristics of lumbar ribs were included in the analysis.

Inclusion criteria:

- original clinical study reporting on the prevalence of lumbar ribs, where the lumbar rib was defined as the accessory rib present in the lumbar vertebrae of the normal spine, i.e., the spine characterised by normal segmentation and quantity of thoracolumbar vertebrae (17).
- outcomes including prevalence, laterality, and anatomical location of the lumbar ribs.

Exclusion criteria:

- inappropriate publication type (i.e., meta-analysis, review, case report, and conference report).
- studies performed on animals.
- studies performed on fetuses or embryos.
- studies conducted on a group of patients with disorders known to be associated with rib abnormalities, such as Williams-Beuren syndrome or childhood cancer population.
- lack of clear acknowledgement of the presence of lumbar ribs.
- no information about the segmentation of the spine and the quantity of thoracolumbar, thoracic, and lumbar vertebrae.
- no clear indication of counting the entire vertebral column which would preclude miscalculations of vertebral segmentation in cases of caudal/cranial vertebral shifts.

### Data items and collection process

The data extraction process has been carried out by 2 independent reviewers (M.O. and M.P.). Data regarding the type of the study, year, study modality, country of origin, sample size, incidence of LR, characteristics of population, laterality, site of pathology and gender distribution was obtained. Moreover, symptoms associated with lumbar ribs or other clinical implications were also noted when possible. In case of any inconsistency or incomplete data the authors of the original article were contacted for clarification and additional details.

### Study risk of bias assessment

Assessment of the quality of included studies has been made using the Aqua Tool [22], which is helpful in determining risk of bias. Evaluation took place in five domains: (1) objective(s) and subject characteristics, (2) study design, (3) methodology characterization, (4) descriptive anatomy, and (5) reporting of results; and each domain was classified as either ,,Low”, ,,High” or ,,Unclear” risk of bias. When a ,,No” response was given to any signaling question under any of the categories, the domain was deemed to have a ,,High” risk of bias, whereas all ,,Yes” answers indicated a ,,Low” risk of bias. When a clear examination of the study was not possible due to incoherent data, the ,,Unclear” option was selected.

### Effect measures, synthesis methods and certainty assessment

The statistical analysis was conducted by M.O. utilizing MetaXL 5.3 by EpiGear International Pty Ltd. (Wilston, Queensland, Australia) to calculate pooled prevalence estimates of LR. A random-effects model was used for all analyses. Chi-square test and the I^2^ statistic were employed to assess the heterogeneity of the included studies. A *p* value < 0.10 was considered to indicate significant heterogeneity between studies when using chi-square test; for the I^2^ statistic the value was interpreted as follows: 0-40% might not be important; 30-60% could indicate moderate heterogeneity; 50-90% could indicate substantial heterogeneity; and 75-100% could represent considerable heterogeneity [25].

Subgroup analysis was performed based on geographical region, study modality and laterality of LR to analyze the source of potential heterogeneity. Confidence intervals (95%CI) were compared between the groups to determine statistically significant differences, and if they overlapped the difference between the groups was considered statistically insignificant [21]. A leave-one-out analysis was used to evaluate sensitivity in order to further investigate possible sources of heterogeneity.

### Reporting bias assessment

To evaluate the possibility of a small study effect indicating publication bias, a Doi plot with the LFK index was employed [17]. According to the interpretation of the LFK index, absolute values falling between 0 and 1 indicated no significant asymmetry (no significant small-study effect); those falling between 1 and 2 indicated minor asymmetry (might suggest small-study effect); and values exceeding 2 indicated major asymmetry (strongly suggesting presence of small-study effect). Forest and Doi plots were created using MetaXL version 5.3.

## Results

### Risk of bias in studies

Most of the studies included in this meta-analysis were found to be at ,,Low” risk of bias in all domains except for the first domain (objective(s) and subject characteristics), in which more studies are considered to be at ,,High” risk. The reason for that is primarily due to missing demographic data about research groups. In other domains, such as study design, methodology characterization, descriptive anatomy and reporting of results, the vast majority of studies were classified as being at ,,Low” risk of bias. Evaluation of the AQUA tool [22] can be found in Table 1.

### Reporting bias

Small-study effect analysis revealed LFK index values of -4.55 (major asymmetry). (Supplementary material 2)

### Study selection

**Fig. 1:**
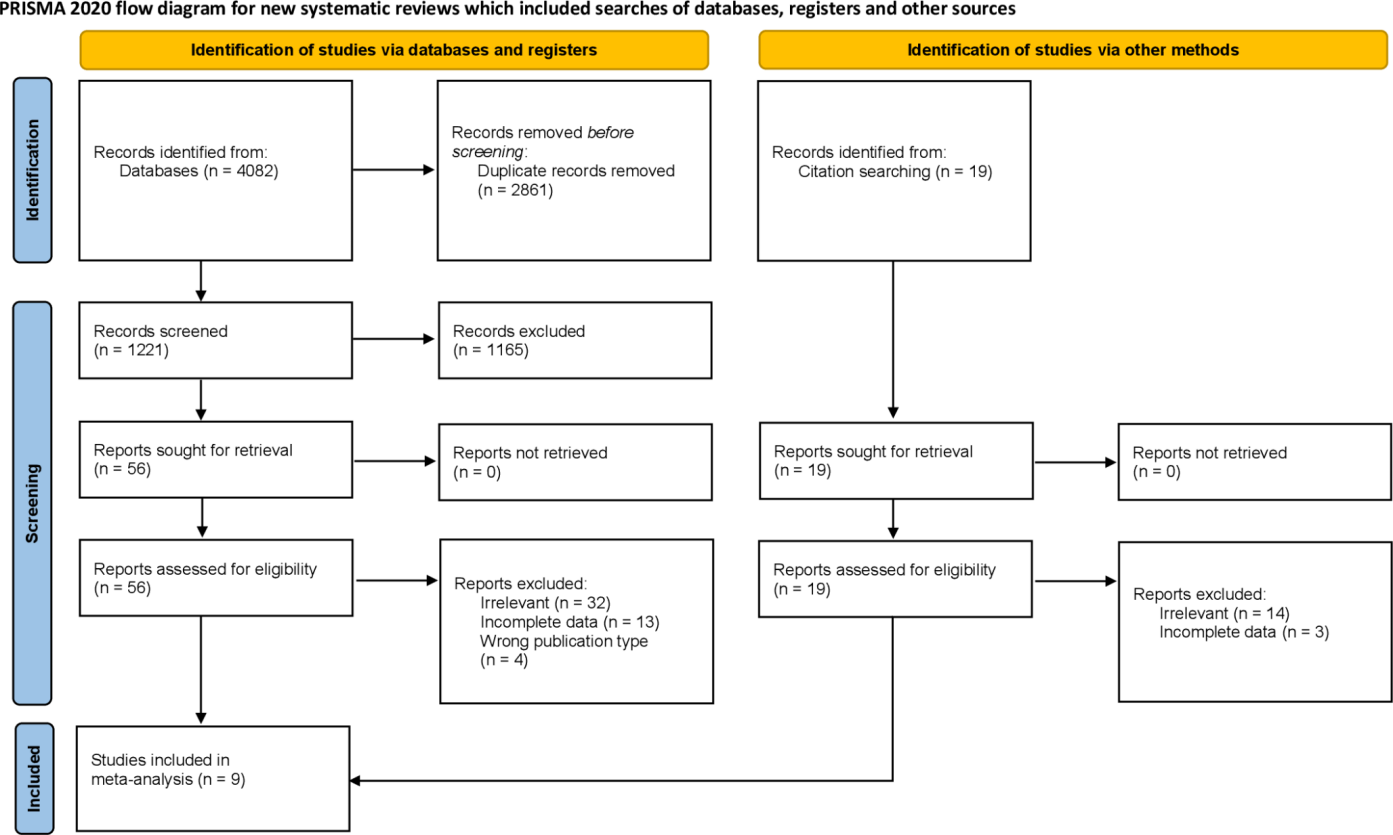
Preferred Reporting Items for Systematic and Meta-analyses (PRISMA) reporting guideline flowchart [36]

Figure 1 describes the study identification process. The initial search of databases resulted in 4082 records. Additionally, 19 studies were identified by the search of the references of the included studies. After removing duplicates and excluding studies that did not meet eligibility criteria, 56 articles were assessed by full text. Finally, a total of 9 [1, 14–16, 20, 24, 32, 33, 39] studies were included in the meta-analysis.

### Study characteristics and results of individual studies

Table 1 provides a summary of the included studies’ characteristics. Overall, 9 studies (5430 individuals) [1, 14–16, 20, 24, 32, 33, 39] were included in the analysis. The research came from 8 countries involving North America, Europe, Asia, Australia and was published between 1930 and 2023. The most frequent type of imaging modality was computed tomography (CT) which was used in 4 studies [14, 15, 33, 39], then Xray (3 studies) [1, 20, 33], while ultrasonography (USG) [24] and magnetic resonance imaging (MRI) [16] was the least common (1 study each). Only one included study [16] enrolled a symptomatic group of patients (patients who underwent lumbar spine MRI for lower back pain or radiculopathy). Rest of the studies generally involved retrospective analysis of imaging data from consecutive patients for a variety of indications.

**Table 1:** Characteristics of included studies and evaluation of their risk of bias by AQUA tool [22]

Study first author & year	Country	Type of study	Number of patients	Number of lumbar ribs (prevalence in %)	Risk of bias - AQUA tool				
					Objective(s) and study characteristics	Study design	Methodology characterization	Descriptive anatomy	Reporting of results
Abul2023 [1]	Turkey	Xray	187	2 (1.1)	low	low	low	low	low
Davran2017 [14]	Turkey	CT	530	7 (1.3)	high	low	low	low	low
Doo2020 [15]	South Korea	CT	1340	5 (0.4)	low	low	low	low	low
Hershkovitz2008 [24]	Israel	USG	367	3 (0.8)	high	low	low	low	high
Merks2005 [32]	Netherlands	Xray	881	8 (0.9)	high	high	low	low	high
Nakajima2014 [33]	Japan	CT	226	13 (5.8)	low	low	low	low	low
Singer1990 [39]	Australia	CT	810	15 (1.9)	high	low	high	low	low
Farshad-amacker2014 [16]	USA	MRI	57	8 (14.0)	low	low	low	low	low
Heise1930 [20]	Germany	Xray	1032	80 (7.8)	high	high	high	high	high

### Results of syntheses and certainty of evidence

#### General prevalence

**Fig. 2:**
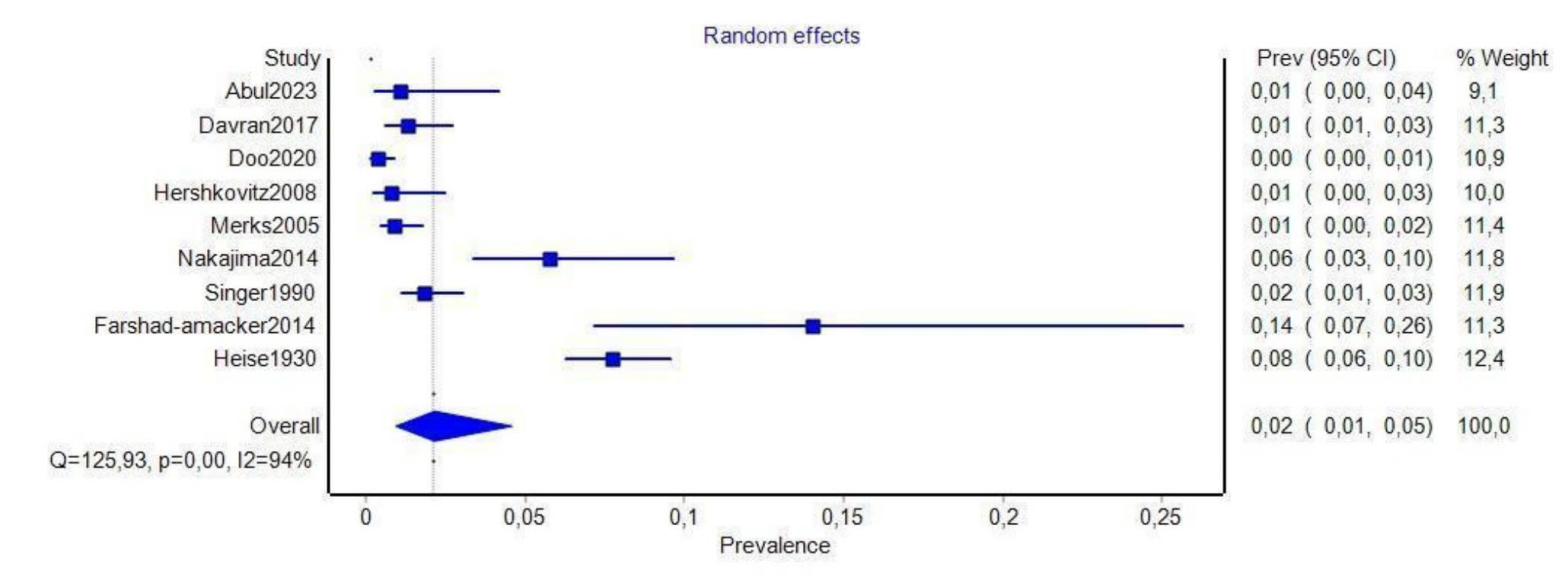
Pooled prevalence estimate of lumbar ribs, forest plot

A total of nine studies [1, 14–16, 20, 24, 32, 33, 39] (*n* = 5430 patients) reported the prevalence of LR (Table 1). Lumbar ribs were found in 141 cases. The pooled prevalence estimate (PPE) of LR was 2.1% (95%CI: 1.0-4.6), which is shown in Fig. 2.

### Prevalence in asymptomatic group of patients

A subgroup analysis involving only studies that included consecutive patients for various indications was conducted. In this analysis, we excluded those studies in which individuals had imaging tests done due to low back pain or radiculopathy that could disturb results as patients sought medical help due to a potential underlying pathology of the spine. Therefore, eight studies [1, 14, 15, 20, 24, 32, 33, 39] (*n* = 5373) were analyzed, and PPE of LR was 1.6% (95%CI: 0.7–3.8; I² = 93.89; Cochran’s Q, p-value = 114.53), which was less prevalent than the general prevalence, however the difference was not statistically significant.

### Prevalence based on types of imaging modality

A total of 5006 patients from seven studies [1, 14, 15, 20, 32, 33, 39] were included in the subanalysis with respect to imaging modality (Table 2), 2906 of them were examined by CT scan and 2100 by Xray. The prevalence of LR in the CT scan group was 1.6% (95%CI: 0.6–4.3) and 2.1% (95%CI: 0.4–11.1) in the Xray group. No statistically significant differences were observed between these two groups.

**Table 2:** Results of lumbar ribs prevalence based on imaging modality

Type of imaging modality proceeded	Number of studies (number of patients)	Number of lumbar ribs	Prevalence (95%CI)	I²: %	Cochran’s Q, *p*-value
CT	4 (2906)	40	1.6% (0.6–4.3)	90.11	30.32
Xray	3 (2100)	90	2.1% (0.4–11.1)	95.20	41.66

### Geographical distribution

Subgroup analysis of seven studies [1, 14, 15, 20, 24, 32, 33] according to geographical distribution of LR (Table 3) included 4563 patients from three regions of the world. The highest prevalence of LR was observed in Europe (2.8%; 95%CI: 3.0–20.0) then in East Asia (1.5%; 95%CI: 1.0-19.2) and Middle East (1.1%; 95%CI: 0.6–20.0), although the differences were not statistically significant.

**Table 3:** Prevalence of lumbar ribs based on geographic region

Geographic region	Number of studies (number of patients)	Number of lumbar ribs	Prevalence (95%CI)	I²: %	Cochran’s Q, *p*-value
East Asia	2 (1566)	18	1.5% (1.0-19.2)	96.38	27.59
Middle East	3 (1084)	12	1.1% (0.6–20.0)	< 0.001	0.5
Europe	2 (1913)	88	2.8% (3.0–20.0)	97.16	37.15

### Laterality of lumbar ribs

Data regarding the laterality of LR was found in 4 studies [14, 24, 33, 39] involving 38 individuals (Table 4). Lumbar ribs were found more frequently bilaterally (24 cases, 65.4%, 95%CI: 39.4–84.6) than unilaterally (14 cases, 34.6%, 95%CI: 15.4–60.6), however the difference was not statistically significant.

**Table 4:** Results of lumbar ribs prevalence based on their laterality

Type of laterality	Prevalence of lumbar ribs % (95%CI)	I²: %	Cochran’s Q, *p*-value
Unilateral	34.6 (15.4–60.6)	45.74	5.53
Bilateral	65.4 (39.4–84.6)	45.74	5.53

## Discussion

A rather uncommon deformity of the lumbar spine, LR can range in appearance from fully developed to rudimentary, additional ribs. Lumbar ribs most commonly originate from the first lumbar vertebra [5], but they can also develop from the second [34] and third lumbar vertebrae [13]. There is currently a lack of studies systematically analyzing the anatomical and clinical considerations, and the prevalence of LR. Thus, the purpose of our research was to conduct a meta-analysis in order to standardize the data regarding the prevalence of LR with geographical considerations. The results of this meta-analysis comprising 5430 patients revealed that lumbar ribs are present in 2.1% (95%CI: 1.0-4.6) of the general population and in 1.6% (95%CI: 0.7–3.8) of asymptomatic individuals. To our knowledge, this is the first study summarizing the data about lumbar rib prevalence, and furthermore, the only one including patients from various geographical regions, which makes the results more applicable to the general world population.

### Anatomical characteristics and differentiation

Differentiating the ,,extra rib” from ,,rudimentary rib” is difficult and reliant on the type of imaging used, however there still is an ongoing debate in the literature on where to set the limit. Wéry et al. [46] distinguished the difference between them based on the length in relation to the proceeding rib. Typically, LR are not longer than half the length of the 12th ribs [4], they also tend to differ from thoracic ribs by their course, which is generally more horizontal and tapers upward as it ends distantly [5]. Aly et al. [4] stated that the anatomical composition of the lumbar rib is as well defined as a “normal rib”, implicating that it differs from the “true rib”. The distal end of the LR has a cartilaginous segment and an ossification center with an absent cartilage [5], and unlike in “normal ribs”, it does not disappear postnatally [4]. Moreover, further studies concluded that both the proximal and distal ends of LR are predominantly non-ossified with a fibrous character [31].

Another issue with differentiating LR can be encountered in spines with abnormal segmentation. Recent cadaver-based study by Ishiguro et al. [27] revealed that abnormal thoracolumbar segmentation of the spine occurred in 5.4% of individuals, with mutations in axial vertebral count constituting 4.5% of them, and lumbosacral “trade-off” (7 C–12T–4–6 S) making for 0.9% of cases. Additionally, out of 105 cadavers included in Ishiguro’s study, 2 individuals exhibited thoracolumbar “trade-off” (7 C–11T–6 L–5 S) [27]. Abnormalities in vertebrae quantity and vertebral “trade-offs” (i.e., cranial and/or caudal vertebral shifts) can significantly alter the physician’s ability to correctly count and label the vertebrae (especially if utilized imaging modality covers only the thoracolumbar segment, and not the whole spine), which may further lead to misdiagnosis concerning the presence of LR (as the number of false positives or negatives will be significantly higher in abnormal spines when contrasted to cases with normal spine segmentation).

### Genetic and developmental factors

Several theories propose the process of supernumerary (SNR) rib formation, especially LR [4]. Although the exact etiology of LR still remains uncertain, multiple researchers hypothesize that the maternal stress in the embryogenesis period along with basic alterations in gene expression may be crucial factors [4], as they develop in places where normally the transverse processes are formed [3]. Mahajan et al. [31] have proposed that inadequate union of the cranial and rostral portions of the sclerotome during embryonic development may be the cause of the LR formation. According to Wéry et al. [46], extra ribs may be an indication of the somites’ changing positional identities as the axial skeleton develops. Compared to fetuses, adults are speculated to have lower incidence of supernumerary ribs, as suggested by Chernoff and Rogers [12], probably due to fetal SNR being made up mostly by ossification centers that may disappear postnatally. Though Tanaka et al. [42] reported the prevalence of lumbar accessory ossification centers (LAOC) between 4% and 7% in developmental stage of XIX to XXIII Streeter’s horizon, and other similar study [35] found 3 cases of LAOC among 136 screened embryos, we decided not to include research done on embryos in this meta-analysis. Furthermore, Bagnall et al. [6] examined fetuses ranging in conceptual age from 8 to 26 weeks obtained from hysterotomies or spontaneous abortions and found 8 out of 728 cases to have additional ossification centers in the lumbar region. Yet, there is a need for more research determining the number of those embryonic additional ossification centers that may develop into fully formed LR. Numerous studies in the literature also indicate a possible connection between the development of LR and genetics [4]. Homeobox (HOX) genes are essential for the development of the axial skeleton in vertebrates [4], and as a result, specific knockouts of distinct HOX genes have been hypothesized to result in spinal congenital malformations. According to Wellik and Cappechi [45], in mice, the Hox11 group gene is specifically necessary for sacral and caudal vertebrae formation, and overexpression of the Hox11 group gene is predicted to cause sacralization or caudalization at various segments of the axial skeleton. Additionally, in the absence of Hox10 function, vertebrae in the lumbar region of the spine are not formed, and instead, ectopic ribs project caudally from thoracic to sacral spinal segments [10, 45]. Gorski et al. [19] concluded that deficiencies in FGD1 signaling directly caused the skeletal abnormalities and were especially responsible for the development of LR.

With an incidence of 1 in 7,500 live births, Williams-Beuren syndrome is an uncommon multisystemic genetic disorder and is associated with a hemizygous microdeletion of chromosome 7 (7q23.11) [37]. In their study, Schmitz et al. [37] found that in the cohort of 91 patients with Williams-Beuren syndrome, 67 (74%) patients were found to have LR, of which in 85% of cases LR were presented bilaterally. Interestingly, lumbosacral transitional vertebrae (LSTV) were present in almost half of the patients, who also demonstrated a higher prevalence of LR, suggesting a possible correlation between those two anatomical variations [37]. Similar results and conclusions were reported by Nakajima et al. [33], as their study also presented a correlation between LSTV and LR. Various authors tried to investigate the concept of exposure to chemical agents as a possible cause of LR development, with strong evidence of bromoxynil and valproic acid association to induce lumbar rib formation [29]. The clinical manifestation of LR is still not precisely understood. Taslimi and Glass [43] demonstrated the association between the trisomy of the 9th chromosome and the development of LR. Aly et al. [4] and Schmitz et al. [37] reported that similarly to Williams-Beuren syndrome, the occurrence of LR can be observed in other disorders: Aarskog syndrome, cleidocranial dysplasia, trisomy 8, Turner syndrome, and incontinentia pigmenti [4, 37, 46]. Thus, the prenatal identification of LR may aid with the diagnosis of aforementioned syndromal disorders [9, 37]. Moreover, several researchers tried to investigate the association between the co-occurence of supernumerary ribs and childhood cancer (especially acute myeloid leukemia, renal tumors and hepatoblastomas) [30, 32, 38, 48], however, the consensus for LR still remain uncertain, as some authors indicate that this connection is only limited to CR [32, 38]. Thus, further research investigating this correlation must be conducted. Anatomical studies further investigating the link between LR, LSTV and CR are also needed, as well as focusing on gender-based and geographical-based subanalyses, as the current literature lacks sufficient information regarding this topic.

### Symptomatic associations with lumbar ribs

Role of LR in generating back pain has been outlined in many cases, but no study unambiguously has proven this association to exist. According to Steiner [40], when analyzing 38,105 roentgenograms he discovered that 12 of the 17 cases of LR observed are clinically associated with symptoms, which were described as severe pain in the back (8 patients), soreness or backache (3 patients) and catching pain in his back (1 patient). In 1926, John Cumming documented a lumbar rib fracture that resulted from the third lumbar vertebra’s transverse process, which fused with the fourth lumbar vertebra’s transverse process on the same side [13]. Ceasar and Carcamo [8] reported another case of a 53-year old male who presented with chronic chest pain after a minor thoracic trauma injury. Subsequently, a careful examination of the back revealed a tender point corresponding to the transverse process of the first lumbar vertebra [8]. A computed tomography scan later showed a lumbar rib arising from the right transverse process of the first lumbar vertebra [8]. The authors hypothesized that the pain resulted from the pseudoarticulation of the lumbar rib innervated by the lateral branch of lumbar posterior ramus of the spinal nerve [8]. The patient then achieved an immediate pain relief following the series of radiofrequency ablations of the pain-source pseudoarticulation of lumbar rib [8]. In contrast, Anap et al. [5] described a case of a symptomatic LR originating from the first lumbar vertebra, without a history of any form of trauma, causing back pain and radiating pain in the left groin and thigh. Additionally, in this case [5] it was demonstrated that LR were the cause of the persistent mechanical irritation of adjacent kidney, which manifested as a pain along the renal region and thus it was proposed by the authors to be labeled as a DASK Syndrome. Interestingly, in another study included in our analysis [16], the investigation of the presence of LR was performed in symptomatic sample of patients (suffering from lumbar back pain or radiculopathy), which showed a distinctively higher prevalence of LR in comparison to remaining studies based on the asymptomatic sample of patients. This implies that further research is needed to analyze the suggested relationship between back pain and LR and moreover, that it is worth considering such an anatomical malformation while diagnosing patients with backache of unknown origin.

### Comparison with cervical ribs

Cervical ribs are one of the oldest recognized anatomic variations and were first described in the second century AD [2]. This anomaly has been reported more frequently than LR and drawn more attention from researchers, because of their association with thoracic outlet syndrome or symptoms such as ipsilateral limb pain, weakness, numbness, or cold intolerance [12, 23]. According to a recent meta-analysis, Henry et al. [23] reported that the prevalence of CR in healthy individuals was 1.1% (95%CI: 0.9–1.4). Additionally, CR are more frequently observed in females than in males (1.3% and 0.7%, respectively); slightly more than half of patients had unilateral CR (51.9%), and no statistically significant differences were observed in CR occurrence between the left and right sides of the spine [23]. Previous studies indicated that CR appear to be more prevalent than LR [3, 4]. Our study does not support this hypothesis, as our results show that the prevalence of LR is higher in comparison to the prevalence of CR, which was reported by Henry et al. [23]. Our results also show that unilateral LR is observed only in approximately every third patient (34.6%, 95%CI: 15.4–60.6). In terms of contrast, Henry et al. [23] demonstrated that CR presents a more even distribution of unilateral and bilateral cases. Due to a lack of sufficient evidence, our study was unable to assess the prevalence of LR in gender-based subgroups. Also, we suggest that the comparison of the prevalence estimates of LR from our study with the prevalence estimates of CR from the study performed by Henry et al. [23] has to be interpreted with caution, as there is a substantial difference in the number of studies included in those meta-analyses (9 studies and 37 studies, respectively), which may introduce bias.

### Regional and ethnic differences in prevalence

A slight but noteworthy diversity was observed in the subgroup analysis of the prevalence of LR according to regional origin. The highest prevalence of LR was noted in the European population (2.8%, 95%CI: 0.3–20.0), then in East Asia (1.5%, 95%CI: 0.1–19.2), and then in the Middle East (1.1%, 95%CI: 0.6–2.0). Our results did not show statistically significant differences between the aforementioned geographical regions. Moreover, the generalizability of these results is subject to serious limitations, as each subgroup includes a low number of studies (2 studies, 2 studies, and 3 studies, respectively), which explains rather wide confidence intervals. In the meta-analysis performed by Henry et al. [23], subgroup analyses on the prevalence of CR according to place of origin revealed a substantial amount of difference, suggesting a potential ethnic predisposition and additionally validating the critical role of genetics in the development of CR.

### Clinical ımplications

Most often, LR are asymptomatic and incidental discovery during imaging studies. According to Aly et al. [4], spiral CT is the preferred diagnostic modality. Our results did not reveal statistically significant differences in the prevalence of LR between studies based on X-rays and those based on CT as a preferred imaging modality. In the past, several researchers reported cases where LR have been mistaken for abnormalities of the abdominal vessels, kissing osteophytes and fractures of the transverse processes [5, 41]. Moreover, Aly et al. [4] mentioned that especially on X-rays, LR may be easily mistaken for pleural lesions, bony lesions or lung consolidations. Additionally, in a clinical practice, it is hypothesized that the presence of LR may pose a considerable challenge during percutaneous renal biopsy through the renal angle, which is typically formed between the last, 12th rib and the first lumbar vertebra [11, 41]. Therefore, the surgeon’s awareness and understanding of LR will aid in the development of a different strategy for nephrectomy or renal biopsy treatments [41].

As previously mentioned, Nakajima et al. [33] demonstrated that the presence of LR is strongly associated with the presence of LSTV, especially sacralisation of the fifth lumbar vertebra, and emphasized that the co-existence of LR with LSTV is shown to be problematic not only in terms of misdiagnosing these two pathologies, but also being the source of miscalculation and misinterpretation of the lumbar spinal segmentation. When advanced sacralisation or lumbarisation is present in a lumbosacral junction along with LR, it might be really challenging to determine the correct spinal level and might impact accurate identifying of each vertebrae depending on the modality used. The lumbar spinal structure may be regarded as typical when LR and lumbarization are present, particularly on lumbar spinal radiographs [33]. Additionally, LR can seriously impair the correct identification of the lumbar vertebrae, as in case of the presence of LR without the co-occurence of LSTV, the normal lumbar spinal configuration may be misinterpreted as sacralisation [33]. A failure in the successful identification of spinal anomalies like LR and LSTV with its subsequent miscalculation of vertebral level may result in serious consequences, such as incorrect level spinal surgery. Malanga and Cook [18] reported a case of a patient in whom neglection of S1 lumbarization resulted in wrong-level emergency decompression related to cauda equina syndrome in a disc surgery. Consequently, according to Bron et al. [7], similar obstacles may be encountered during the administration of epidural or intradural anesthetics in the lumbar area of the spine. Doo et al. [15] noticed that categorization of LSTV is different depending on the counting method used during radiological assessment when a lumbar rib is present. In such a situation, without imaging the entire spine, if the lowest ribs are interpreted as 12th, one patient was given a sacralization diagnosis [15]. However, by counting inferiorly from first cervical vertebrae (C1), the lowest ribs were confirmed as 13th ribs and the patient was diagnosed with lumbarization [15]. All of the above concludes that spinal lumbar abnormalities may not be adequately assessed by lumbar radiograph examination alone and highlights the importance of whole-spine imaging, which is the only technique that guarantees the correct numbering of thoracic and lumbar vertebrae in all cases and remains the gold standard.

### Limitations and future directions

Our analysis was limited by the significant degree of variability in the included research. We attempted to look into the source of the heterogeneity using subgroup analysis, but it persisted throughout the whole investigation process. Furthermore, a small-study effect analysis with an LFK index value of -4.55 indicated a significant asymmetry in the Doi plot for the investigation of the overall prevalence of LR. This finding suggests that there may be a negative publication bias, meaning that studies with lower LR prevalence rates have a better chance of being published than those with higher prevalence rates. The study protocol was not previously registered for this systematic review and meta-analysis, which is another disadvantage. The worldwide survey discovered that although this protocol is recommended, it is not done commonly [44]. The absence of studies carried out in Australia, Oceania, South America, and Africa, as well as a low number of studies in the analyzed subgroups, may restrict the generalizability of the findings. Although we were able to identify individual case reports of back pain associated with LR, it was not enough to analyze this relation and therefore further research is needed.

## Conclusions

According to our study, lumbar ribs are more common than previously thought and share a prevalence of 2.1% in general. Lumbar ribs are in majority of cases incidental findings and mostly asymptomatic, however, in some patients, they might be associated with back pain. The presence of lumbar ribs may be associated with various spinal congenital anomalies, such as cervical ribs and lumbosacral transitional vertebrae, as well as syndromes like Williams-Beuren syndrome, Aarskog syndrome, and Turner syndrome. Moreover, the knowledge on the prevalence and anatomy of lumbar ribs is crucial for spinal surgeons to avoid wrong level surgery. Importantly, according to Hu et al. [26], 50% of spine surgeons acknowledged to perform a wrong-level surgery at least once in their medical career. In this sense, LR awareness is especially critical to preventing not only spinal surgical malpractice, but also to avoid unnecessary complications during anesthetic or renal procedures.

## Electronic supplementary material

Below is the link to the electronic supplementary material.

Supplementary Material 1

Supplementary Material 2

## Data Availability

The manuscript’s data was obtained from the original publications. The corresponding author can provide the complete set of data used in this analysis upon reasonable request.
