# Supplementary figures and images for: Prevalence and characteristics of lumbar ribs: a meta-analysis with anatomical and clinical considerations
Source: Surg Radiol Anat. 2024 Oct 8;46(12):2057–66. doi: 10.1007/s00276-024-03504-9 (PMC11579145; doi:10.1007/s00276-024-03504-9)

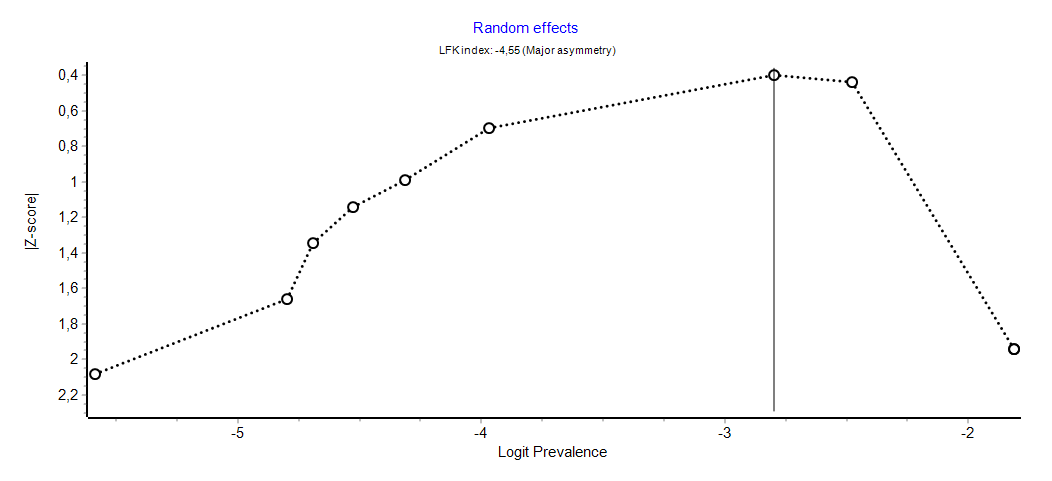

Supplement: Supplementary file 2 — Supplementary Material 2 [file 276_2024_3504_MOESM2_ESM.tiff]
